# Signal transduction growth factors: the effective governance of transcription and cellular adhesion in cancer invasion

**DOI:** 10.18632/oncotarget.16300

**Published:** 2017-03-16

**Authors:** Marina Di Domenico, Antonio Giordano

**Affiliations:** ^1^ Department of Biochemistry, Biophysics and General Pathology, University of Campania “Luigi Vanvitelli”, Naples, Italy; ^2^ IRCCS Institute of Women's Health Malzoni Clinic, Avellino, Italy; ^3^ Department of Medicine, Surgery and Neuroscience, University of Siena, Siena, Italy; ^4^ Sbarro Institute for Cancer Research and Molecular Medicine, Center for Biotechnology, Temple University, Philadelphia, PA, USA

**Keywords:** cancer invasion, e-cadherin, phosphorylation, EGFR TGFβ, KGF

## Abstract

Giulio Bizzozero classified the tissues concerning their capacity to self-renew during the adult life in labile, stable and permanent tissues. In 1940 Viktor Hamburger and Rita Levi Montalcini exposed the possibility to induce the growth of permanent cells thanks to a specific ligand Nerve Growth Factor (NGF). Stanley Cohen purified a protein the Epidermal Growth Factor (EGF), able to induce epidermis proliferation and to elicit precocious eye disclosure and teeth eruption, establishing the “inverse” relationships between the proliferation and differentiation. These two biological effects induced by EGF were according to EGFR signaling is involved in a large array of cellular functions such as proliferation, survival, adhesion, migration and differentiation. This review is focused on the key role of growth factors signaling and their downstream effectors in physiological and in pathological phenomena, the authors highlight the governance of Growth factors during the EMT in cancer invasion.

## INTRODUCTION

Stanley Cohen described on “The Journal of Biological Chemistry” a protein called Epidermal Growth Factor (EGF) able to induce epidermis proliferation and to elicit precocious eye disclosure and teeth eruption [1]. Some years later it was discovered that radioactivity of labelled EGF may be localized in many tissues (e.g. epidermis, corneal epithelium, liver, mammary gland) so it was described an EGF pleyotropic action [2–5]; just 30 years ago on 1986 the Nobel Committee proclaimed Stanley Cohen and Rita Levi-Montalcini, the first scientist who discovered the Nerve Growth Factor, winners of the prize for Medicine and Physiology.

**Figure 1:**
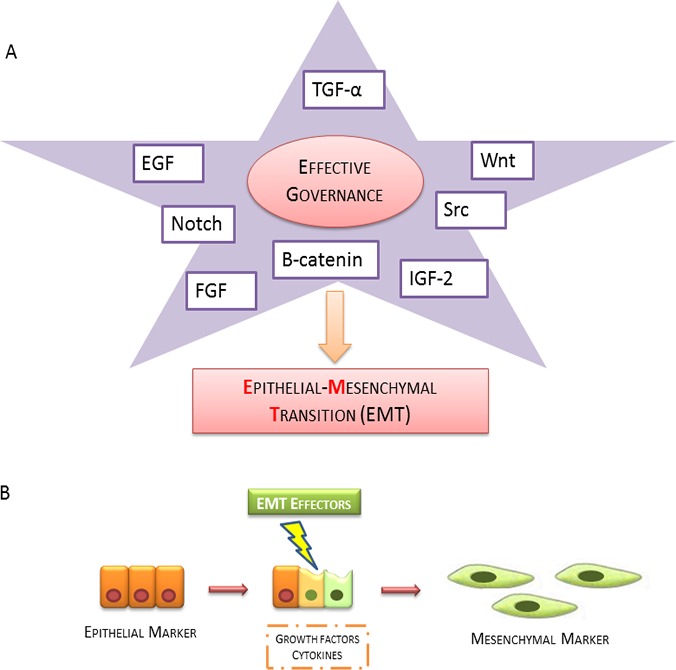
Epithelial mesenchymal transition scheme **A.** The broad spectrum of growth factors and related activating downstream effectors (EGF, TGF-α, WNT, Notch, Src, β-catenin, IGF-2 and FGF) involved in induction of EMT **B.** Epithelial cells promote the expression of mesenchymal markers by the production of growth factors cytokines.

The interaction between epithelial and stromal cells is crucial for differentiation and epithelial growth in the course of morphogenesis, embryonal stage and in wound healing of various tissues [6, 7]. A part of growth factors, such as the Epidermal growth factor (EGF), Fibroblast growth factor (FGF), Transforming growth factor-alpha (TGF-α), Keratinocyte growth factor (KGF) and Insulin-like growth factor 2 (IGF2y) can induce, both morphogenesis in different organisms as well as, the development and growth of many tumor entities. Most oncogenic pathways mediated by peptide mitogens FGF, EGF, TGF-α and IGF2y, and Src, Ras, Ets, Integrin, Wnt/b-catenin and Notch signalings, can promote Epithelial Mesenchymal Transition (EMT) [8] (Figure 1A-1B). Cadherins genes activated in embryogenesis are also activated during carcinoma progression and metastasis, indicating that EMT involves a reactivation during tumor progression. Growth factors activity overlaps with the Cadherinsreported to be involved in EMT. The elucidation of the various processes that modulate Cadherins and Growth Factor Receptors (GFR) signal transduction, like receptor heterodimerization and endocytosis, has highlightened new therapeutic opportunities and focused the mechanisms contributing to the effectiveness of existing anticancer treatments [9]. The involvement of cadherins is recognized in the normal epithelial tissue organization and in cancer promoting as discussed previously [10]. The authors describe that metastatic phenotype and EMT are typically linked together with E-cadherin down-regulation [11], and that the interplay between Growth Factor Receptors and cadherins promotes tumorigenesis. The aim of this review is focused on the key role of growth factors signaling and its downstream effectors promoting various biological events. The authors purpose is to emphasize the governance of growth factor receptors in progression and cancer invasion.

## CROSS-TALK BETWEEN EPITHELIAL GROWTH FACTOR RECEPTORS AND STEROID RECEPTORS IN EMT

In humans, more than 30 ligands of the group of the EGF receptors (EGFR) are located, as a multi-layered signal-transduction network, at the beginning of a complex. The interaction between EGFR and its ligand leads to an EGFR monomer dimerization or with another member of erbB family, then these events mediates tyrosine kinase activation. Ligand dependent or independent activation of receptor tyrosine kinases (RTKs) can support EMT signaling in normal and epithelial cells, but finally that cell migration and invasion depend additionally upon i) which receptor heterodimers are assembled ii) which downstream targets are activated iii) how RTKs signaling influences cellular adhesion and matrix ECM interactions in tissue environment [12].

The EGFR has two domains, an extracellular domain that accept the ligands and an intracellular TK domain; these two domains are connected by a transmembrane hydrophobic peptide region. EGFR is a glycoprotein with a molecular weight of 170-kD. EGFR ligands are betacellulin, EGF, amphiregulin, TGF-α, heparin, and epiregulin [13].

**Figure 2:**
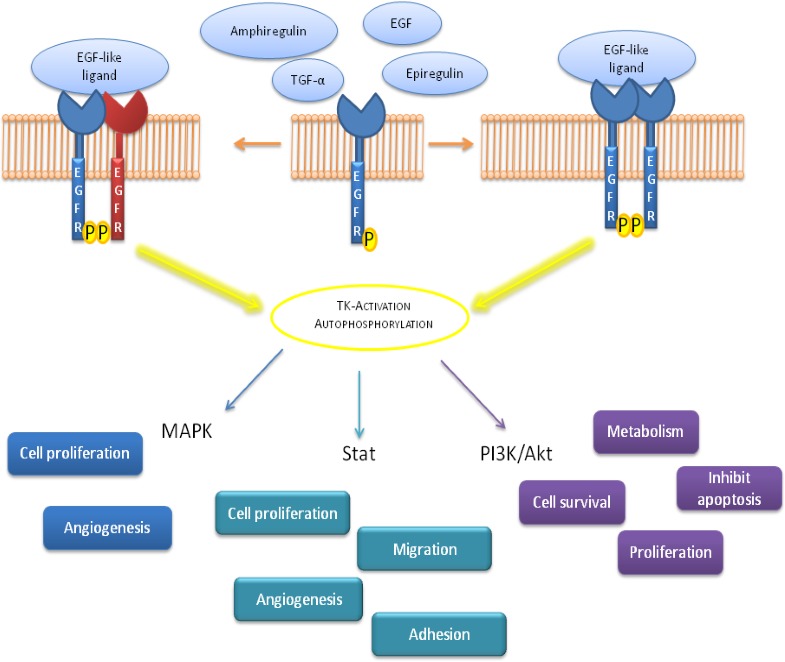
Schematic representation of signal transduction pathways involved after EGFR activation EGFR ligands binding leads to a receptor dimerization followed by Tyrosine Kinase (TK) autophosphorylation; this mechanism induces a down-stream precise signaling correlated to different biological effects.

**Figure 3:**
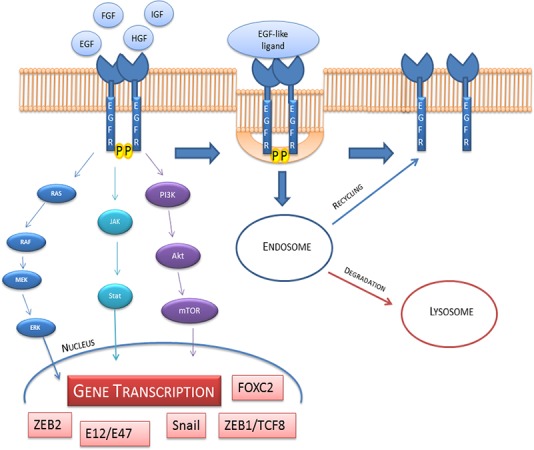
EGFR activation, internalization, recycling and degradation The down-stream signaling involve Ras/Raf/Mek/Erk; Jak/Stat and PI3K/Akt/mTOR pathways. These pathways activate the transcription of ZEB1/TCF8, Snail, ZEB2, Snail2, E12/E47, FOXC2 that are able to influence the cell fate. On the right side of the figure we show the internalization and endosomal sorting of EGFR.

EGFR is localized in its monomeric form in the cell membrane, upon ligand binding, EGFR dimerization leads to Tyrosine Kinase (TK) activation and autophosphorylation on tyrosine residues. These phosphorilations are crucial steps indownstream physiologic and pathogenic events [14]. The binding of EGFR-ligand can activate various signaling pathways, main pathways comprise PI-3K/Akt and MAPK ways. The Ras-Raf-MEK-MAPK signaling prevents apoptosis, induces cell proliferation and the release of angiogenetic factors and promote metastasis, while PI-3K/Akt pathway regulates cell surviving, metabolic effects, proliferation and apoptosis (Figure 2). The described signaling is “turned off” by endocytosis of EGFR/ligand. Upon activation, EGFR is translocated and transported to endosome and it is either reprocessed back to the plasma membrane or degraded in lysosomes (Figure 3) [15, 16].

EGFR signaling activation is triggered by the phosphorylation of the intrinsic kinase receptor domain and this event contributes to the recruitment of different substrates [17]. This binding is allowed by Src homology 2 motifs (SH2) included on tyrosine kinase domains. On following disruption, the related adaptors and signaling cascades effectors, which consist of KRAS-BRAF-MEK-ERK, phosphoinositide 3-kinase (PI3K), phospholipase C gamma protein, the anti-apoptotic AKT kinase and the STAT, are activated and lead to angiogenesis, survival and cancer promoting [18–23]. Numerous mutations related to different genes implicated in described targets signaling are on the basis of this biological event.

Transcription factors such as ZEB1/TCF8, Snail, ZEB2, Snail2, E12/E47, FOXC2 are involved in growth factors signaling [21] (Figure 3) and it is triggered by the induction of anti-apoptotic signals via receptors such as EGFR, Fibroblast growth factor receptor (FGFR), Platelet derived growth factor receptor (PDGFR), Keratinocytes growth factor receptor (KGFR), cMET, Transforming growth factor β receptor (TGFβR), Human growth factor receptor (HGFR) and Akt, mTOR which are PI3K down-effectors. In order to demonstrate tumor motility, strong evidences in literature proposed the involvement of Snail, Snail2 and E2a transcription factors to explain the disruption of cell adhesion [8].

Modified transduction pathways, in which it was described a crosstalk between EGFRs and steroid receptors have been depicted in the progression of human tumors. An EGFR up-regulation has been represented in a large range of tumors, characterized more aggressive phenotype of these cancers compared to those with a low or normal expression [24–26]. The steroid ligand,17b-Oestradiol (E2)is able to transiently up-regulate EGFR mRNA and protein both in human breast cancer cells and in the rat uterus [27, 28]. The amount of appropriate levels of heterodimers between the two Estrogen Receptor (ER) isoforms mediates estrogenic effects through synergistic mechanism, these results are shown also by others authors in a different systems [29].

Zuo et al. using two human Basal phenotype breast cancers (BPBC) type with respect to Estrogen Receptor/Androgen Receptor ER/AR cell lines as model, have underlined a refractory hormone Progesterone Receptor pathway that is involved in EMT mechanisms through mPRα, Cav-1, EGFR, and PI3K/Akt mediators [30]. mPRα receptor is able to modulate on caveolar membrane of BPBC cells. It promotes EGFR signaling and related inhibition of PI3K pathway and EMT. Others authors shown that steroid receptors contributed to enhance EGF signaling, and Src dependent-EGFR tyrosine phosphorylation in mammary and prostate cancer cells [31]. The interplay between steroid receptors and EGF receptor results in the up-regulation of EGFR tyrosine phosphorylation by EGF in Cos cells transfected transiently with ERα and AR. This phosphorylation determined the assembly of the AR/ER/Src complex [31]. The EGFR signaling regulates the balance between proliferation, apoptosis, differentation and neoplastic development and progression of different human carcinomas. EGFR regulates focal stromal cells associated with invasion and migration in physiological and neoplastic epithelial cells [32] .

Salvatori et al.demonstrated a dose dependent activation of the EGFR gene expression by ligand-bound ERα [33]. To explain this mode of action they described a model using the induction the EGFR gene expression by estrogens was dependent upon the formation of a ERα-Sp1. This model have shown a mechanism in which ERα enhances EGFR gene transcription through protein-protein interactions that substitutes DNA binding. The ERα target is considered to be the transcription factor Sp1, which contributes to the stabilization of an Sp1-dependent initiation complex on a TATA-free template, through enhancing the interaction of Sp1 to its site [34].

These authors have shown, in another report, a relevant dependent gene expression for ERα, that was revealed when ERβ transcriptional ability was tested on the minimal promoter. The sensitivity of target cells to estrogens and selective estradiol receptor modulators (SERMS) can be affected by the two different isoforms of the ER, depending on the formation of appropriate amount and ratio of heterodimers [34].

## KERATINOCYTE GROWTH FACTOR AND FIBROBLAST GROWTH FACTOR PATHWAYS

KGFR was described in CaCo-2 cells (intestinal epithelium) during spontaneous differentiation *in vitro* [35]. Many evidences demonstrated that functional KGFRs, in monolayer cells, have a plasma membranes distribution in a basolateral polarized manner and KGFRs are up-regulated during the beginning of differentiation induced by cell confluence. During differentiation, the KGFR expressing cells are not responsive to EGFR ligands [35, 36]. Another experimental study suggested that KGFs factors have a role of estromedin-like in mammary cancer progression. KGF-13 short peptides are suggested as possible therapeutic application for their biological features [37, 38]. FGFR is involved in various physiological processes during embryogenesis. Four related genes belong to the EGFRs gene family; FGFR1 and FGFR2 encoded during homeostasis of adult tissues [39]. Two splicing transcript variants are encoded by FGFR2. These variants were discovered in mesenchymal tissue, while KGFR was found in epithelial tissue [40]. D'Amici et al. proposed a clear model in which FGFR2 is modulated by cytokines and forms a paracrine loop together with KGFR. Inflammatory cytochines such as interleukin (IL 1β e IL6, Inf-6, Inf-γ and TNF-α) are involved in EMT [41, 42].

**Figure 4:**
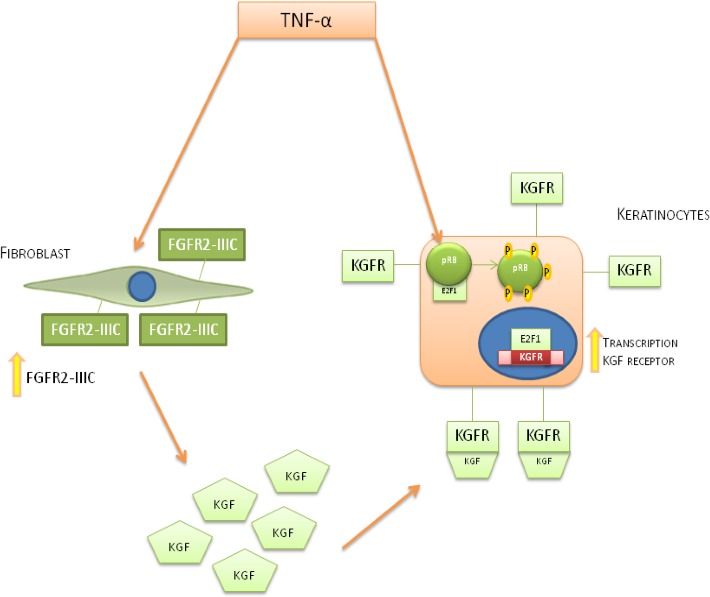
Mechanism of action of TNF-α on fibroblast cells and keratinocytes cells TNF-α stimulation induces an increase of KGF biosynthesis in fibroblast cells and FGFR2-III expression and the expression of KGFR on keratynocites cells. The interplay between KGF and KGFR on keratinocytes leads to an hyper-phosphorylation of pRb, inducing the release of transcriptionally active E2F1 allowing KGFR biosynthesis.

The described mechanism of action by D'Amici et al.have shown a way of TNF-α on both fibroblasts and keratinocytes at the site of inflammation. In this way, fibroblasts increase the expression of FGFR-IIIc on their surface and enhance the production of KGF followed by this ligand binding and activating its own receptor which is also stimulated by TNF-α. In keratinocytes TNF-α induces phosphorylation of pRb and therefore E2F interacts with FGFR2 promoter in order to induce KGFR biosynthesis(Figure 4) [3, 42–45].

## OTHERS PATHWAYS CORRELATED TO EMT

### Notch pathway

Notch signaling pathway is involved both in physiological that in pathological EMT processes [46].

The activation of Notch signaling pathway is able to stimulate tumorigenesis via regulating EMT [47].

It is known, that in mammals, the Notch family consists of 4 receptors allocated on the membrane:Notch1-Notch4 and its 5 ligands: Jagged-1, Jagged-2, Delta-1, Delta-3, and Delta-4. Das I et al. proposed an interplay between EGF and Notch, in fact Notch receptors are characterized by an extracellular domain and an intracellular domains: RAM, Ankyrin repeats, and a C-terminal PEST [48].

Notch gene overexpression in immortalized endothelial cells leads to EMT by Snail activation and VE-cadherin repression [49, 50].

The complex of Notch ligands/Notch receptors induce the cleavage of intracellular domain of Notch (NIC) by c-secretase. In order to realize a complex with RBP-Jk/CBF1, Su(H), Lag-2 and mastermind-like, Notch intracellular domain is translocated into the nucleus [51–53].

Understanding the molecular mechanism of Notch pathway suggests new rational approaches for cancer therapy [54].

The Notch signaling activation induce the expression of different targets involved in cellular proliferation, such as Cyclin D1 and surviving gamma-secretase inhibitors prevent oxaliplatin-stimulated activation of Notch-1 signaling contributing to the increase of chemo sensitivity in colon cancer cells [55].

Notch-1 overexpression was found in T-cell leukemias and Notch1 pathway is activated in different tumors such as lung adenocarcinoma [56].

Oskarsson and Joan Massague [57] have shown that pulmonary metastatic niches providethe survival of disseminated tumor cells (DTCs) in different tissues. The involved molecular components such as Notch-1 and WNT represent a novelty to develop target therapies to target DTCs. Fiorelli A et al.isolated Circulating cells from peripheral blood, in order to propose a validmarker in the diagnostic workup of lung lesions [58].

Notch is considered a key tumor suppressor gene and its genetic alterations lead to atypical pathway activation [59, 60].

### Wnt/β catenin pathway

Wnt's family includes 19 glycoproteins that have a governance position in the control of different biological events, including self-renewal, polarity, motility and cell fate in stem cells. Wnt/β catenin signaling are activated by interaction with Frizzled (FZD) family receptors. The pathway is induced by ligand-receptor complex, the stimulation from outside of the cell to the internal surface activate Disheveled (DVL) which can induce a multitude of downstream target proteins. This protein prevents β-catenin degradation. In addition Wnt regulates calcium dependent and small-GTPases, such as RAC, RhoA, and CD42 [61, 62].

Another class of FZD molecules, the Wnt receptors ROR1 and ROR 2 induce tumor progression [63]. During the EMT the signaling Wnt/β-catenin complex depends by SLUG activation which translocates into the nucleus for different functions after the interaction with β-catenin. Slug cooperates with TCF/LEF [64], and enhances the target gene transcription [65]. Others receptors such as PTK7, VANG and CELSR bind Wnt receptors to control planar cell polarity (PCP) pathways in vertebrates [66].

Wnt/ β-catenin is involved in regulation of physiological and pathological processes [67].

**Figure 5:**
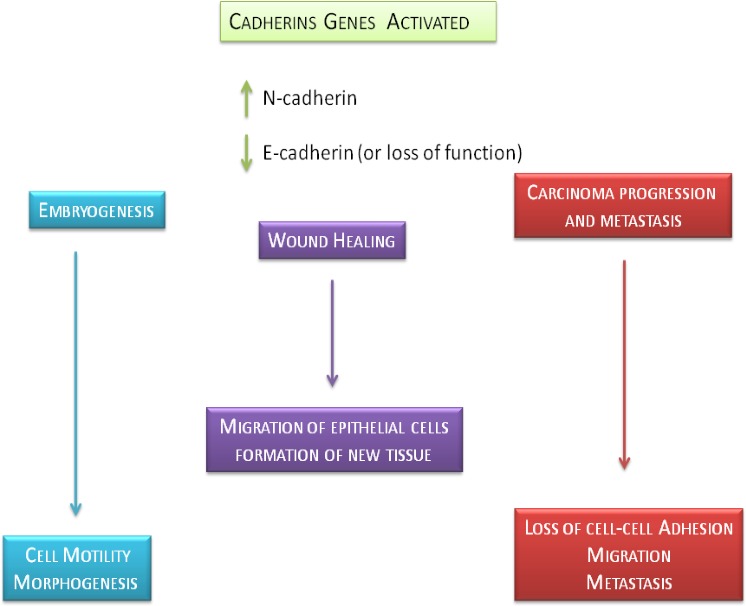
Physiological and pathological role of E-cadherin and N-cadherin genes This figure shows the precise role of E-cadherin and N-cadherin in physiological and pathological biological effects such as embryogenesis, wound healing and carcinoma progression and metastasis.

Wnt/β-catenin activation promotes EMT during gastrulation [68], controls EMT in zebrafish [69, 70] and when deregulated, leads to an early EMT in the mouse [50, 71]. Wnt/β-catenin induces EMT during progression of colorectal cancer metastasis and squamous cell carcinoma (SCC) progression (Figure 5) [72, 73].

### Cadherins in epithelial mesenchymal transition

During embryogenesis, E-cadherin goes through spatial and temporal changes of the which allow the occurrence of cell movement and morphogenesis [74]. It induces a migration of epithelial cells on the surface of the wound with the formation of new tissue [75]. In tumors, the cell-cell adhesion is often weak, giving the cells the possibility to promote metastasis. E-cadherin controls cell motility as “invasion suppressor” [76, 77]. The expression of cadherins, in particular of E-cadherin, has been studied in different human tumors, such as cancers of the prostate, breast, pancreas, lung, cervix, liver, stomach, colon, bladder and squamous cell carcinoma [78]. The neoplastic cells show the E-cadherin loss or can maintain an high expression forming possible cell aggregates [79]. Poorly differentiated cancers show a large expression of E-cadherin without keeping its function, probably due to alterations in the complex of catenin [80, 81]or due to mutation of the catenin [82]. In general, however, the low expression of E-cadherin is described in aggressive phenotype cancers characterized by significant cellular infiltration [83–87]. These evidences are confirmed by *in vitro* assays in which the E-cadherin transfected cells prevent tumorigenesis, therefore deficit in E-cadherin is increasing cell adhesiveness and down-regulating EGFR [77, 88, 89]. Conversely, the knockdown on E-cadherin and β-catenin using siRNA induces an invasive phenotype in epithelial cells [90]. Expression of E-cadherin is correlated to an increased incidence of metastases in lymphatic tissue in different cancers [91]. The mouse ovary carcinoma cells, strongly expressing E-cadherin, show a low metastatic potential [92]. However, no correlation has been described between the presence of E-cadherin and gastric adenocarcinoma and colon metastasis [93].

The role of cadherins in the progression of cancer is much more complex than it was thought at the beginning. The function of E-cadherin is related to the interaction with catenins sites juxta-membrane to form functional units often called catenin-E-cadherin units [94]. The loss of the expression of surface E-cadherin was correlated with a loss of some molecules in lymph node metastases of breast cancer [95]. A previous study has shown that lymph node metastases of breast cancer have a re-expression of E-cadherin or catenins, suggesting that the loss may not be necessary for the metastasis [94].

The E-cadherin is also involved in the regulation of different proteases. Indeed, the E-cadherin transfection in cells prostate cancer E-cadherin-negative line caused a reducing of the production of metalloproteinase MMP-2 and a concomitant reduction of invasiveness [96]. In a similar manner, the cell-cell contacts mediated by E-cadherin involving in the down-regulation of the expression of MMP-9 in skin SCC [97].

In several carcinomas, inappropriate N-cadherins expression has been shown to contribute to a migratory phenotype [98, 99] and Katafiasz et al. have shown that in breast cancer cells the N-cadherin promotes motility independently from the expression of E-cadherin [100]. There is also increasing evidence of coordinate regulation or “cross-talk” between different families of adhesion molecules so that the sniff in the functional capacity of a class of receptors (eg. cadherins) has an impact on another class of receptors (integrins). Weaver et al and Fournier et al. have observed that variants of tumorigenic cell lines of breast cancer could be induced to re-express functional E-cadherin using antibodies blocking the β1 integrins [101, 102]. It was also noted that the cross-talk between integrins and E-cadherins complex and IGF-1 promote migration in colon cancer cells inducing adherence and signal transduction [103, 104]. Finally, increased circulating E-cadherins have been recognized in patients with gastric and hepatocellular cancer [105, 106] and also in several patients with skin cancer, suggesting a possible role as biomarker in certain malignancies [107]. In conclusion E-cadherin ability to maintain the epithelium stratified integrity is lost in more aggressive cancers, such as oral carcinomas [108, 109], because there is a reduction in E-cadherins expressions.

## CROSS-TALK BETWEEN GROWTH FACTOR RECEPTORS AND CADHERINS

Cross-talk between RTKs and cadherins [110, 111] is considered as a pivotal mechanism inducing biological cellular events in tumor environment. Kyriakakis et al. have demonstrated that an atypical glycosylphosphatidylinositol (GPI)-anchored T-cadherin (T-cad) is considered as a negative regulator of EGFR signaling in A431 cells [112, 113]. After EGF induction, T-cad was reallocated to cellular adhesion where it interacts with Tyr1068 phosphorylated EGFR. Cellular adhesion of T-cad relocalization induced by EGF is prevented by EGFR inhibitors such as gefitinib or lapatinib filipin, actin microfilament polymerization (cytochalasin D or cytochalasin B), p38MAPK (SB203580) or Rac1 (compound4). These data demonstrate that T-cad translocation is responsive of EGFR transactivation. EGFR and T-cad in concert represents a novelty in EGF activation pathway [113]. Another recent experimental study reports that the promoter methylation status of E-cadherin gene (CDH1) in Oral Squamous Cancer cells is correlated with E-cadherin protein expression in a large statistical evaluations, and that this finding correlated with patient outcome. In this contest, it was reported that EGFR participates to a bidirectional cross-talk with E-cadherin [114, 115]. It was described that EGFR phosphorylation enhances its internalization into endocytosis vesicles together with e-cadherin. Here, the EGF interacts with its membrane receptor and its activation induces co-localization of E-cadherin and EGF-R in the cytoplasm of cancer cells [109].

## EPITHELIAL MESENCHYMAL TRANSITION AND TUMORIGENESIS

Many carcinomas show a different expression of cadherins, although this is often most evident in poorly differentiated tumors or in advancement of the lesions. Some authors have suggested that a loss or a reduction of E-cadherins determines an increase invasion and metastasis in SCC of the head and neck [116]. In this study the expression of E-cadherin appears well correlated with the differentiation of neoplasia, where the poorly differentiated tumors appear to be completely negative. Dayan and Vered have found a similar pattern of reduced expression in poorly differentiated tumors although this was not statistically significant [117]. Tsanget al. have found an up-regulation in the expression of P-cadherin in severe dysplasia and a decrese in E-cadherin expression and/or cadherins in situ carcinomas and infiltrating tumors [118]. They concluded that the destruction of the complex E-cadherin-catenin is a tardive result correlated with tumor infiltration. Some authors have found that the tumors shown a heterogeneous distribution of E-cadherin and that the loss of E-cadherin shows a statistically significant correlation with the degree of oral cancer or with lesions in the advanced stage and nodal metastasis [119, 120]. In contrast, others authors did not show a link between tumor differentiation and E-cadherin expression [121, 122]. In oral squamous cell carcinoma was described a correlation between tumor stage and E-cadherin and integrin expression, instead P-cadherin expression is mutable [123]. Their results concluded that in all SCC, there could be the precence of cell adhesion molecules and that the loss of integrins and E-cadherin are strictly associated. Harada et al., have observed a desmosomial cadherin in oral cells carcinoma and its low expression in undifferentiated tumors, while more recently they correlated the overexpressed desmosomalcadherins to the loss of invasiveness in SCC cells. [124, 125]. An oral SCC cell lines *in vitro* study has shown a reduced cadherins expression during tumorigenesis [126]. Schipper et al. shown that differentiated tumor lymph node metastatic sites were E-cadherin negative [127]. In opposition with these results, Bowie et al. have shown the same pattern of staining between lymph node metastatic tumors and primary tumors. Sorscher et al. and Hirao et al. obtained similar results [91, 128, 129].

The expression of E-cadherin is considered as tumor predictor biomarker. Yamada et al. have connected the E-cadherin reduction to tumor advanced stages and poor survival [130]. Mattijssen et al. have underlined the correlation between E-cadherin expression and patient survival while Bowie et al. not found this correlation [131].

**Figure 6:**
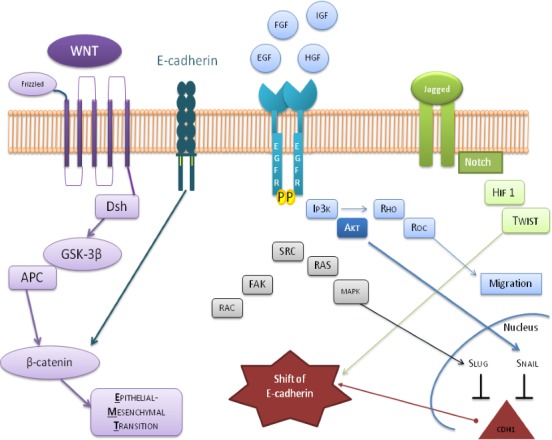
Summary diagram of positive or negative feedback circuits that regulate key processes during EMT The critical crosstalk between important oncogenic factors, and tumor suppressive proteins are represented by the involved pathways which consist of the activation of WNT by β-catenin, the involvement of PI3K and MAPK in response to EGF ligands and HGF, describes the expression of Jagged-1 (JAG1), induced by Notch ligand in the regulation of Snail/Slug and Twist at transcriptional, translational, and posttranslational levels. All these effectors converge on the shift of E-cadherin.

Islam et al. recognized an inadequate expression of N-cadherin in a fibroblastic-like phenotype SCC cell line [132]. In this cell line there is a E-and P-cadherin down-regulation. Transfection of this cell line with an antisense N-cadherin led to the reversion toward apparently normal squamous epithelial cells with increased expression of E-and P-cadherin. In a similar manner, the transfection of a squamous epithelial cell, apparently normal, with the N-cadherin resulted in a fibroblastic phenotype and a down-regulation of E-and P-cadherin. In this review it was discussed about a distint epigenetic modification of DNA, which promotes methylation-induced gene silencing, has been described in a large number of tumors, and includes an increasing number of tumor suppressors. Infact aberrant methylation of genomic DNA are partly responsible for transcriptional silencing during carcinogenesis. Recent evidence indicates that the loss of the protein E-cadherin (wich has a key role in adhesion and tissue formation) due to epigenetic aberrations, including the hypermethylation of the promoter of the gene coding for itself, has a crucial role in the onset of progression triggering cancer invasion and metastasis in various human cancers [133]. The gene coding mutations for the E-cadherin is the gene CDH1 (cadherin 1, type 1) located on chromosome 16q22.1 [134], have already been described with regard to carcinomas of the head and neck [135, 136]; even though most of the studies were limited to small sample sizes, in short follow-up, or incomplete information of the treatment of the patient for which it was not possible to formulate a definitive statement about carcinogenesis (Figure 6).

Recently Kabashima-Niibe et al. have demonstrated that also mesenchymal stem cells are implicatedin tumor-promoting cancer stroma development and in the regulation of epithelial-mesenchymal transition in pancreatic tumor cells [137]. Mesenchymal stem cells are multipotent adult stem cells, often used in numerous clinical trials. MSCs are isolated from a wide of tissue and organs such as adipose tissue [138–140], it's generally acknowledged that their plasticity properties allow them, in response to specific stimuli, to transdifferentiate into mesodermal lineagescells which include smooth, skeletal and cardiac muscle and to be committed towards a cardiac-like phenotype. [141–144].

Battula et al. have demonstrated that EMT-derived cells and MSCs are characterized by an MSCs antigenic profile; moreover MSCs express Twist, Snail (EMT-associated genes) and mesenchyme forkhead 1 (FOXC2). In addition, functional analyses have revealed that EMT-derived cells can differentiate, as MSCs, into chondrocytes, osteoblasts and adipocytes. These authors shown that EMT-derived cells are able to invade MDA-MB-231 breast cancer cells comparable to MSCs [145].

## CONCLUSIONS

The understanding of biological mechanisms associated with cancer could contribute to isolate new targets for novel therapeutic interventions.

It's known that EMT is induced by growth factors and cytokines signals released from the neoplastic microenvironment including [146–148]. Cells undergoing EMT lose their epithelial morphology, reorganize their membrane ruffling and gain motility. The up- and down-regulation of several molecules including RTKs, steroid receptors, cadherins and their correlate pathways can represent useful target for cancer treatment [149].
